# Influence of Passivation Layers on Positive Gate Bias-Stress Stability of Amorphous InGaZnO Thin-Film Transistors

**DOI:** 10.3390/mi9110603

**Published:** 2018-11-17

**Authors:** Yan Zhou, Chengyuan Dong

**Affiliations:** Department of Electronic Engineering, Shanghai Jiao Tong University, Shanghai 200240, China; zhou_yan@sjtu.edu.cn

**Keywords:** amorphous InGaZnO (a-IGZO), thin-film transistor (TFT), positive gate bias stress (PGBS), passivation layer, characteristic length

## Abstract

Passivation (PV) layers could effectively improve the positive gate bias-stress (PGBS) stability of amorphous InGaZnO (a-IGZO) thin-film transistors (TFTs), whereas the related physical mechanism remains unclear. In this study, SiO_2_ or Al_2_O_3_ films with different thicknesses were used to passivate the a-IGZO TFTs, making the devices more stable during PGBS tests. With the increase in PV layer thickness, the PGBS stability of a-IGZO TFTs improved due to the stronger barrier effect of the PV layers. When the PV layer thickness was larger than the characteristic length, nearly no threshold voltage shift occurred, indicating that the ambient atmosphere effect rather than the charge trapping dominated the PGBS instability of a-IGZO TFTs in this study. The SiO_2_ PV layers showed a better improvement effect than the Al_2_O_3_ because the former had a smaller characteristic length (~5 nm) than that of the Al_2_O_3_ PV layers (~10 nm).

## 1. Introduction

Amorphous InGaZnO thin-film transistors (a-IGZO TFTs) have considerable potential for applications in next-generation flexible, transparent, and large-size flat panel displays (FPDs) because of their superior electrical characteristics, such as large field-effect mobility (~10 cm^2^/V·s), low subthreshold swing (~0.2 V/decade), small leakage current (<10^−13^ A), and so on [1,2]. However, the reliability issues, e.g., threshold voltage (V_th_) shift under positive gate bias stress (PGBS), remain to be solved. Both charge trapping [3,4,5,6] and ambient atmosphere effect [7,8,9,10,11,12,13,14] have been reported to be responsible for V_th_ shifts in a-IGZO TFTs during PGBS tests. Meanwhile, some research groups have demonstrated that the bulk trapping effect [15,16] and plasma damage [17,18] could also lead to V_th_ shifts under PGBS. Evidently, this V_th_ instability is not preferred. In fact, PGBS instability may seriously hinder the actual applications of a-IGZO TFTs in FPDs because it may directly impact the brightness uniformity and stability of display panels. Therefore, some measures must be taken to make the devices more stable during PGBS tests. Passivation (PV) layers, such as SiO_2_, Si_3_N_4_, etc., have been reported to exhibit a good resistance to ambient atmosphere, and thus improve the PGBS stability of a-IGZO TFTs [19,20,21,22,23,24]. However, the exact physical mechanism for how PV layers make devices more stable remains unclear. In this paper, we sputtered SiO_2_ (or Al_2_O_3_) with different thicknesses to passivate a-IGZO TFTs, observing the variation of their PGBS instability. Both SiO_2_ and Al_2_O_3_ were chosen for the PV layers because of their good compatibility in TFT process integration. It was found that the V_th_ shift was reduced by increasing the PV layer thickness and the SiO_2_ improved the PGBS stability of a-IGZO TFTs more significantly than the Al_2_O_3_. The related physical mechanism was classified based on the experimental observations. 

## 2. Materials and Methods

**Figure 1:**
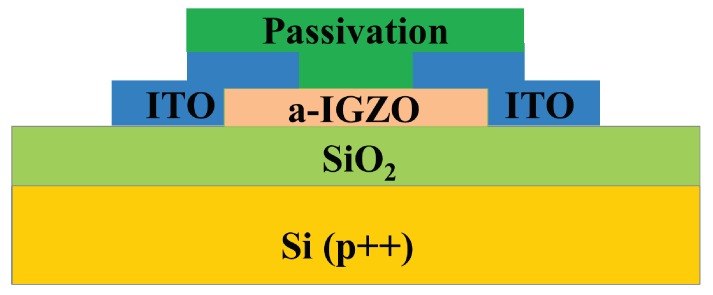
Schematic cross-section of the inverted staggered amorphous InGaZnO (a-IGZO) thin-film transistors (TFTs).

Inverted staggered a-IGZO TFTs were fabricated, the schematic cross-section of which is shown in Figure 1. P-type silicon wafers (gate electrodes) with a 200 nm-thick thermal SiO_2_ (gate insulators) were used as substrates. After thorough cleaning, 50 nm-thick a-IGZO films (In:Ga:Zn = 1:1:1 in mol ratio) as the channel layers were prepared on the substrates using radio frequency (RF) magnetron sputtering at room temperature (RT) with a power of 60 W, a pressure of 5 mTorr, and an Ar flow rate of 30 sccm. Then, Indium Tin Oxide (ITO) films with a thickness of 200 nm were deposited as source/drain (S/D) electrodes using direct current (DC) magnetron sputtering at RT, where the power was 100 W, the pressure was 5 mTorr, and the Ar flow rate was 30 sccm. For the passivated devices, SiO_2_ (or Al_2_O_3_) films with different thicknesses were deposited using RF sputtering at RT with ta power of 50 W, a pressure of 5 mTorr, and an Ar flow rate of 30 sccm. The channel layers, S/D electrodes, and PV layers were patterned using shadow masks during their depositions, leading to a channel width/length (W/L) of 1000/275 μm. Finally, the devices were annealed at 400 °C for 1 h.

The electrical characteristics of the TFTs were measured using a 2636 A parameter analyzer (Keithley Instruments, Inc., Beaverton, OR, USA) in an unsealed chamber, which maintained the atmospheric pressure and little gas circulation. The moisture content in the chamber was controlled by feeding the water molecules with the flow of N_2_. All the devices were measured at RT in darkness. For the transfer curve measurements, V_DS_ of 10 V was employed. In this study, V_th_ is defined as the gate voltage of the normalized drain current (I_DS_/(W/L)) reaching 100 nA.

## 3. Results and Discussion

**Figure 2:**
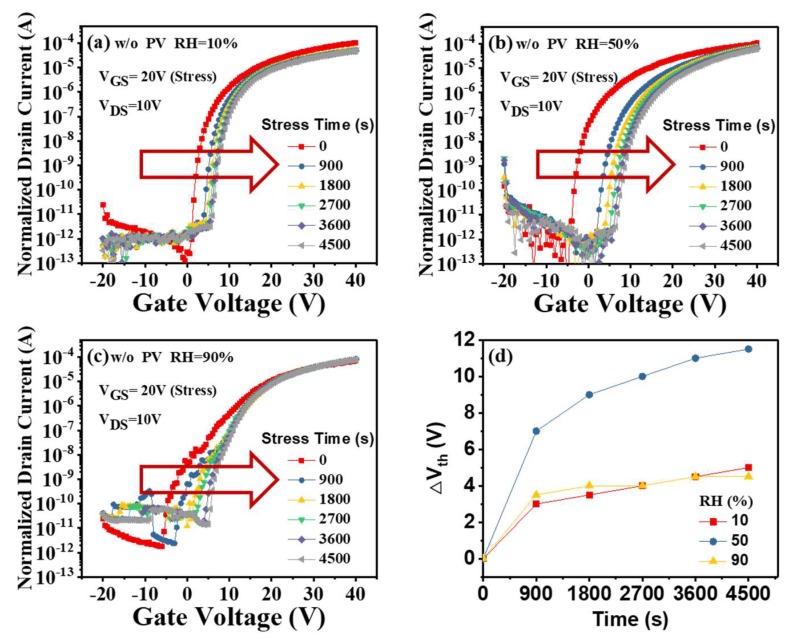
Transfer characteristics of the unpassivated a-IGZO TFTs as a function of the positive gate bias-stress (PGBS) time under relative humidity (RH) of (**a**) 10%, (**b**) 50%, and (**c**) 90%, respectively; (**d**) variations of the ΔV_th_ with PGBS time for the a-IGZO TFT devices.

Figure 2a,c shows the time evolution of the transfer characteristics of the unpassivated a-IGZO TFTs under PGBS as well as the relative humidity (RH) of 10%, 50%, and 90%, respectively. During the PGBS tests, direct voltage of +20 V was applied to the gate electrodes for a period and then the transfer curves were instantly measured. With the increase in the stress time, the transfer curve positively shifted, which was apparently influenced by RH. In order to quantitatively describe the stable properties of a-IGZO TFTs under PGBS, we defined a useful term ΔV_th_, the difference between the V_th_ under stress and its initial value. The ΔV_th_ values under various RH were extracted and listed in Figure 2d. After 4500 s of PGBS test, the positive V_th_ shifts of 5 V, 11.5 V, and 4.5 V were observed under RH = 10%, 50%, and 90%, respectively. It is worth noting that the largest ΔV_th_ occurred at RH = 50% (as shown in Figure 2d), which is consistent with our previous report [22].

The positive V_th_ shift of a-IGZO TFTs under PGBS was attributed to charge trapping at the dielectric/channel interface (front-channel effect) [3,4,5,6], ambient atmosphere effects at the back surface (back-channel effect) [7,8,9,10,11,12,13,14], or bulk trapping in the IGZO bulk (bulk effect) [15,16]. According to our previous work [22], The biggest V_th_ shift at RH = 50% is mainly attributed to the competition of oxygen (or moisture) adsorption/desorption at the IGZO back surface during PGBS tests. This result indicates that RH = 50% is the severest condition to characterize the bias-stress stability of a-IGZO TFTs.

**Figure 3:**
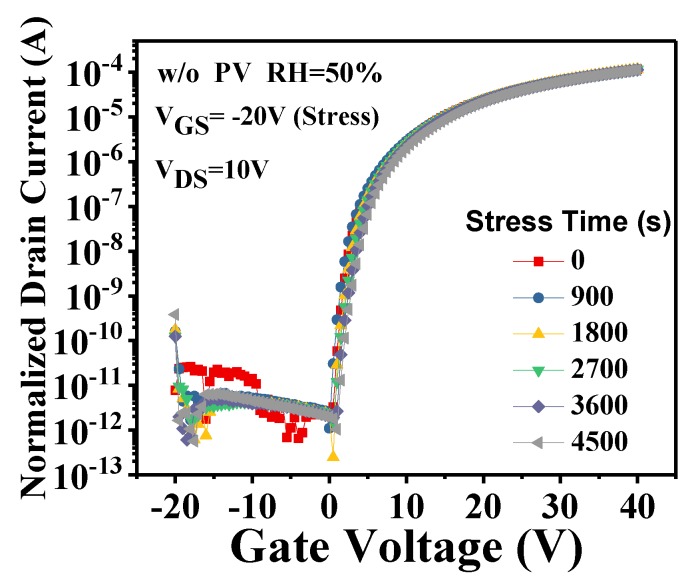
Transfer characteristics of the unpassivated a-IGZO TFTs as a function of the negative gate bias-stress (NGBS) time under RH = 50%.

In addition, we measured the negative gate bias-stress (NGBS) instability of a-IGZO TFTs at RH = 50%, as shown in Figure 3. During the NGBS tests, a direct voltage of −20 V was applied to the gate electrodes for a period and then the transfer curves were instantly measured. After 4500 s of NGBS test, nearly no V_th_ shift was observed. When a negative voltage was applied to the gate electrode of a-IGZO TFTs, the oxygen atoms in a-IGZO tended to be repelled into the ambience, leading to negative shifts of V_th_ [25]. However, this process might have been effectively prohibited by the moisture-assisted oxygen adsorption [11,12,22], especially when the ambient RH was high. Therefore, no evident V_th_ shifts were exhibited during the NGBS tests in this study.

In this study, we deliberately adopted the severest measurement condition (RH = 50%) to examine the influence of PV layers on the bias-stress stability of a-IGZO TFTs. Since the devices were rather stable during the NGBS tests, only PGBS stabilities were characterized for the following studies.

It is well-known that PV layers can effectively improve the stability of TFT devices, whereas the exact physical mechanism involved is still not very clear. However, we may phenomenally describe the dependence of V_th_ shift (ΔV_th_) during PGBS tests on PV layer thickness (d) as follows [19],

(1) $$\Delta V_{\mathrm{th}}=\alpha \cdot e_{\tau }^{d}+\beta$$

where β is the V_th_ shift affected by charge trapping, bulk trapping, and plasma damage, α is a constant relating to the V_th_ shift affected by ambient atmosphere, and ε is the characteristic length related to the gas diffusion. When d is larger than ε, the ambient gases hardly influence the PGBS stability of a-IGZO TFTs. In other words, the characteristic length ε is the critical dimension for the ambient atmosphere effect during PGBS tests. From an application perspective, a small ε is usually preferred.

To further investigate the ambient effects during PGBS tests, the a-IGZO TFTs were applied using PV layers with different thicknesses. SiO_2_, one of the most popular dielectric materials in TFT fabrications, was used to passivate the devices here. For comparison purposes, Al_2_O_3_, another dense material [23], was also adopted as PV layers for the a-IGZO TFTs in this study. The water vapor transmission rate (WVTR) and oxygen transmission rate (OTR) are reported to be inversely proportional to the PV layer thickness [24]. To analyze the influence of PV layers on PGBS stability of a-IGZO TFTs in depth, SiO_2_ and Al_2_O_3_ films with different thicknesses (0–30 nm) were deposited to passivate the devices.

**Figure 4:**
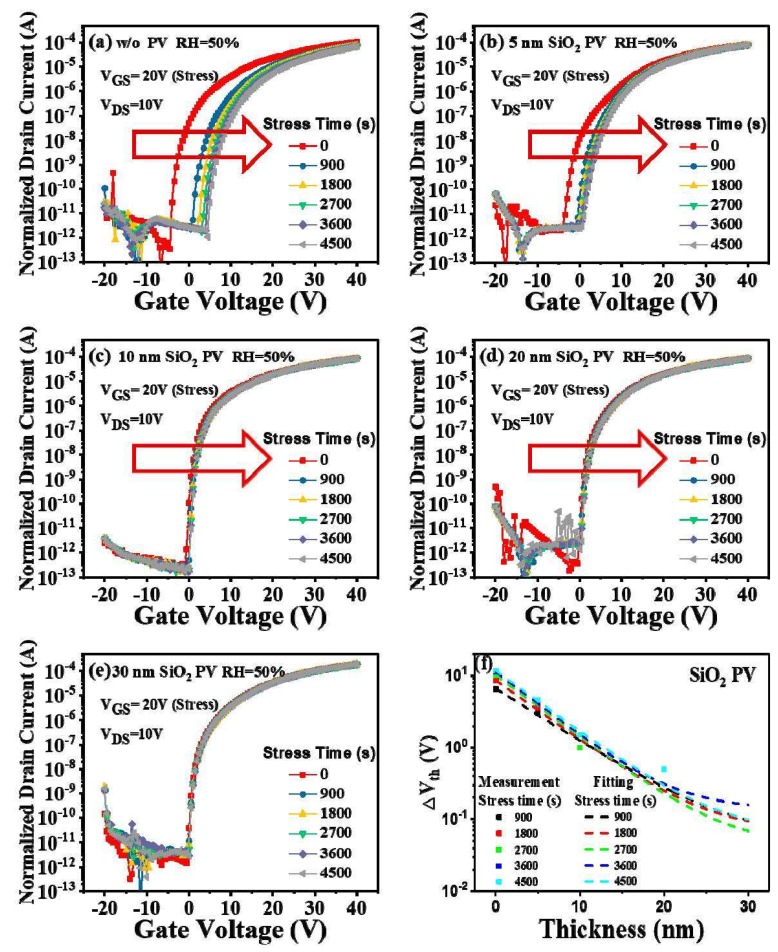
Stress-time dependence of the transfer characteristics of the a-IGZO TFTs with a SiO_2_ PV layer thickness of (**a**) 0 nm, (**b**) 5 nm, (**c**) 10 nm, (**d**) 20 nm, and (**e**) 30 nm, respectively; (**f**) experimental data and fitting curves of the ΔV_th_ under PGBS as a function of PV layer thickness of the a-IGZO TFTs.

Figure 4a,e shows the PGBS time evolution of the transfer characteristics of the a-IGZO TFTs with a SiO_2_ PV layer thickness of 0 nm, 5 nm, 10 nm, 20 nm, and 30 nm, respectively. We noticed that the passivated a-IGZO TFTs exhibited a similar tendency to that of the unpassivated device, i.e., with the increase in the stress time, the transfer curve gradually shifted in the positive direction. However, the a-IGZO TFTs with SiO_2_ PV showed more stable properties during the PGBS tests. To describe this tendency more clearly, we extracted the V_th_ shifts and listed them in Figure 4f. When the PV layer thickness increased from 0 nm to 30 nm, the ΔV_th_ decreased evidently from 12 V to nearly 0.1 V after 4500 s of bias stress test. This can be attributed to the PV layer barrier effect, i.e., preventing the exchange of O_2_/H_2_O molecules between the channel layers and the ambient atmosphere. When the PV layer thickness was larger than 5 nm, the V_th_ of the a-IGZO TFTs barely changed. This can be understood by considering the concept of characteristic length (see (1)) in PV layers, which was about 5 nm here. When the SiO_2_ PV layer thickness was smaller than the characteristic length, the O_2_ molecules easily diffused from the atmosphere into a-IGZO (the H_2_O diffused inversely) under PGBS, resulting in positive V_th_ shifts of the a-IGZO TFTs. As the PV layer thickness was larger than ε, the diffusion of O_2_/H_2_O molecules through the PV layers became rather difficult. This is why the device with a thicker PV layer showed less degradation of its electrical behavior. Since a sufficiently thick PV could nearly eliminate the V_th_ shifts (as shown in Figure 4f), we can assume that the ambient atmosphere effect, rather than charge trapping, dominated the instability of a-IGZO TFTs during the PGBS tests in this study.

**Figure 5:**
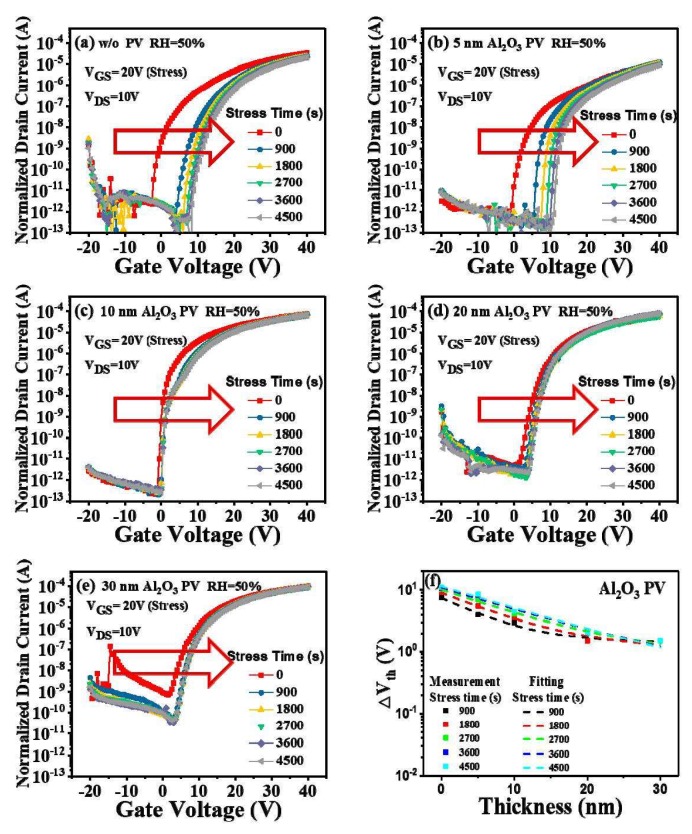
Stress-time dependence of the transfer characteristics of the a-IGZO TFTs with an Al_2_O_3_ PV layer thickness of (**a**) 0 nm, (**b**) 5 nm, (**c**) 10 nm, (**d**) 20 nm, and (**e**) 30 nm, respectively; (**f**) experimental data and fitting curves of the ΔV_th_ under PGBS as a function of the PV layer thickness of the a-IGZO TFTs.

For comparison purposes, we also measured the PGBS stability of the a-IGZO TFTs passivated by Al_2_O_3_ PV layers with a thickness of 0 nm, 5 nm, 10 nm, 20 nm, and 30 nm, respectively, as shown in Figure 5a–e. We may observe that a fairly similar tendency to the case of SiO_2_-passivated devices was obtained here, i.e., the a-IGZO TFTs under PGBS became increasingly stable with the increase in the Al_2_O_3_ PV layer thickness. Meanwhile, for both Al_2_O_3_ and SiO_2_ PVs, the transfer curve positively shifted as the PV layer thickness increased, which can be attributed to the extra interface states generated during the PV depositions [26,27]. However, the Al_2_O_3_ PV layer also exhibited something different. As shown in Figure 5e, for the device with a thick PV layer (≥20 nm), its leakage current gradually rose with the increase in the stress time. This phenomenon was probably due to the plasma bombardment on the surface. Since the deposition rate of Al_2_O_3_ (0.7 nm/min) was smaller than that of SiO_2_ (1.5 nm/min), more sputtering time was needed for the deposition of the Al_2_O_3_ PV layers, leading to more serious plasma damage at the back channels. What is more, the ion bombardment of the plasma can result in a positive V_th_ shift [17,18], which explains why the leakage current of the devices with thick Al_2_O_3_ PV layers increased during the PGBS tests.

To precisely denote the influence of the Al_2_O_3_ PV layer on the PGBS stability of a-IGZO TFTs, the V_th_ shifts were extracted and listed in Figure 5f. Compared with the data shown in Figure 4f, we may note that the Al_2_O_3_ PV layers had an inferior barrier function to SiO_2_. When the Al_2_O_3_ PV layer thickness was larger than 10 nm, the V_th_ shift of the devices changed slightly, indicating that the characteristic length of the Al_2_O_3_ PV layer was around 10 nm. When the PV layer thickness reached 30 nm, the ΔV_th_ became much smaller (~1 V), again confirming that the ambient atmosphere effect dominated during the PGBS tests in this study. 

So far, we have obtained two important experimental results: (1) the PGBS stability of a-IGZO TFTs gradually improved with the increase in PV layer thickness; (2) the SiO_2_ PV layer exhibited a better improvement effect on the PGBS stability than Al_2_O_3_. In order to discuss the theoretical origin of these results, we extracted the critical parameters in (1) of the PV layers. We fit the measurement data of SiO_2_ PV and Al_2_O_3_ PV with (1), as shown in Figure 4f and Figure 5f, respectively. One may observe that the fitting curves agreed well with the measurement data, from which the fitting parameters were obtained and summarized in Table 1.

As shown in Table 1, the α values of both PV layers were much larger than the β values for the same stress time, indicating that the ambient atmosphere effect instead of charge trapping dominated during the PGBS tests in this study. Therefore, with the increase in PV layer thickness, the ambient atmosphere effect was more strongly prevented, resulting in better PGBS stability of a-IGZO TFTs. The α value increased with the increase in the bias time, whereas the ε remained nearly unchanged. The increase in α resulted from more O_2_/H_2_O exchange between the device back channels and the ambience, leading to a larger V_th_ shift. Most importantly, SiO_2_ and Al_2_O_3_ exhibited quite different characteristic length (ε) values, as shown in Table 1. The characteristic length of the SiO_2_ PV layers (~5 nm) was far smaller than that of Al_2_O_3_ (~10 nm), leading to better improvement of the PGBS stability of a-IGZO TFTs by SiO_2_ PV layers than Al_2_O_3_. Therefore, based on our results, the sputtered SiO_2_, rather than the sputtered Al_2_O_3_, should be preferred to passivate a-IGZO TFTs in applications of FPDs.

## 4. Conclusions

The transfer curve of a-IGZO TFTs shifted positively during the PGBS tests, which could effectively be improved by applying PV layers. In this work, both SiO_2_ and Al_2_O_3_ films with different thicknesses were used to passivate the a-IGZO TFTs, indicating that the ambient atmosphere effect rather than charge trapping dominated the V_th_ shifts during the PGBS tests. A simple model was used to theoretically discuss the related physical mechanism. With the increase in PV layer thickness, the devices became increasingly stable, as a result of the stronger prevention of the ambient atmosphere effect. When the PV layer thickness reached the characteristic length, the variation in V_th_ became quite small. The SiO_2_ PV layer showed a better improvement effect than the Al_2_O_3_ PV layer because the former had a smaller characteristic length.

## Figures and Tables

**Table 1:** Fitting parameters of the PV layers used for a-IGZO TFTs.

Materials	Stress Time (s)	α (V)	β (V)	ε (nm)
SiO_2_	900	6.47	0.06	5.93
	1800	8.46	0.07	5.22
	2700	10	0.04	5.03
	3600	10.86	0.14	4.86
	4500	11.45	0.06	5.13
Al_2_O_3_	900	6.07	1.39	9.24
	1800	7.95	1.09	9.53
	2700	9.31	0.84	10.21
	3600	10.46	0.30	11.23
	4500	11.07	0.25	11.06
